# Development of environmental tools for anopheline larval control

**DOI:** 10.1186/1756-3305-4-130

**Published:** 2011-07-06

**Authors:** Susan S Imbahale, Collins K Mweresa, Willem Takken, Wolfgang R Mukabana

**Affiliations:** 1 Laboratory of Entomology, Wageningen University, P.O. Box 8031, 6700 EH Wageningen, The Netherlands; 2 International Centre of Insect Physiology and Ecology, P.O. Box 30772 - 00100 GPO, Nairobi, Kenya; 3 School of Biological Sciences, University of Nairobi, P.O. Box 30197-00100 GPO, Nairobi, Kenya

## Abstract

**Background:**

Malaria mosquitoes spend a considerable part of their life in the aquatic stage, rendering them vulnerable to interventions directed to aquatic habitats. Recent successes of mosquito larval control have been reported using environmental and biological tools. Here, we report the effects of shading by plants and biological control agents on the development and survival of anopheline and culicine mosquito larvae in man-made natural habitats in western Kenya. Trials consisted of environmental manipulation using locally available plants, the introduction of predatory fish and/or the use of *Bacillus thuringiensis *var. *israelensis *(*Bti*) in various combinations.

**Results:**

Man-made habitats provided with shade from different crop species produced significantly fewer larvae than those without shade especially for the malaria vector *Anopheles gambiae*. Larval control of the African malaria mosquito *An. gambiae *and other mosquito species was effective in habitats where both predatory fish and *Bti *were applied, than where the two biological control agents were administered independently.

**Conclusion:**

We conclude that integration of environmental management techniques using shade-providing plants and predatory fish and/or *Bti *are effective and sustainable tools for the control of malaria and other mosquito-borne disease vectors.

## Introduction

Development activities that entail clearing of forests and/or drainage of swamps for timber, agriculture, human settlement and road construction often create suitable breeding sites for malaria mosquitoes [1-4]. Irrigated fields and areas with vegetable crops are ecologically good breeding sites for anopheline larvae [5-8] and they indirectly lead to sustained levels of malaria transmission [9]. The gradual increase in human population in western Kenya has put pressure on land available for farming and as a consequence, areas that were previously natural swamps and forests have been transformed into agricultural fields that provide suitable breeding habitats for mosquitoes.

One way of adapting to changes in land use and preventing the transmission of mosquito-borne disease may be achieved through the control of immature mosquitoes. The control of immature mosquito populations is advantageous because the larvae are usually concentrated, relatively immobile, and occupy a minimal habitat area compared with adults [10,11]. Several larval control programs in China, India and Sri Lanka have shown great success in controlling mosquitoes through good water management practices [12]. In Africa, malaria prevention through the control of immature mosquitoes has not received as much attention as adult mosquito control [11]. This is despite the fact that successful larval control of mosquitoes in Africa by environmental management and application of larval insecticides was reported more than half a century ago [13-15], and that there is renewed interest by the scientific community to assess the feasibility of these methods of disease control [16-20].

Mosquito larval control can be achieved through environmental (water) management, use of insect growth regulators, biological and chemical control [10,21]. Environmental management entails modification and manipulation of the environment, and modification or manipulation of human habitation or behaviour to prevent propagation of mosquito vectors and subsequently reduce human vector pathogen contact [22,13]. However, specifications for environmental management vary with local ecosystem structure, and hence there is no uniform environmental management recipe that is appropriate in all settings [13,23]. Biological control methods directed against mosquitoes mostly refer to the use of natural enemies such as predatory fish, invertebrate predators, and toxins produced by microbial agents [10,21,24-26].

We recently reported that, while members of communities affected by malaria are willing to take part in mosquito control activities [27], there is lack of evidence-based research on locally-applicable strategies. A longitudinal study carried out in these same communities showed that larval populations of *Anopheles gambiae *Giles are continuously present [5]. In the present study we investigated the potential of environmental manipulation (shade from crop and non-crop plants) and biological agents (larvivorous fish and the microbial insecticide *Bacillus thuringiensis *var. *israelensis*) for the control of anopheline mosquito larvae.

## Materials and methods

### Study site

The field study was conducted in Nyalenda (0°06'S and 34°46'E, 1100 m above sea level), a peri-urban, low-income area in Kisumu County, western Kenya. The site represented a swamp transformed to sustain irrigated agriculture. The main economic activities were subsistence agriculture with rice, maize, sweet potatoes and vegetables under cultivation. Commercial nurseries of ornamental plants and trees were also present. The area received a total annual rainfall of 1004 mm and experienced a mean annual relative humidity of 64% and air temperature of 23°C in 2007. The area receives short seasonal rains in the months of October through December, while long rains occur between March and June with year-to-year variation in intensity. Water present at the Nyalenda study site was in parts turbid and polluted with debris and human waste from the adjacent housing estates. Additional experiments were conducted in screen-house at the Centre of Global Health Research (CGHR), KEMRI, Kisian, located 13 km north-west of Kisumu city.

### Mosquito colony

*Anopheles gambiae *Giles *sensu stricto *larvae (Iguhu strain) used in experiments were maintained at the KEMRI insectaries in Kisian. Each larval tray was provided with 100 mg of brewer's yeast daily (Pharmadass Ltd., Harrow, UK).

### Fish colony

A colony of *Gambusia affinis *(Cyprinodontiformes: Poeciliidae) was established from a wild-caught population kindly provided to us by staff of the Kenya Marine and Fisheries Research Institute (KEMFRI), Kisumu County. The mosquito-fish colony was maintained in a screen-house at KEMRI, Kisian. The fish were fed on a locally made fish food supplement obtained from KEMFRI. Adult fish were used for screen-house trials and field experiments.

### Mosquito larval sampling

Larval sampling was done using the standard dipping method with a 350 ml mosquito dipper (Bioquip, Gardena, CA, USA) as described by Service [28]. A maximum of 10 dips were sampled from each habitat. Sampled larvae were identified based on morphological characteristics, counted and classified as anophelines and culicines. The larvae were recorded either as early instars (L1 and L2) or late instars (L3 and L4) and mosquito density was expressed as number of larvae per dip. Late instar anopheline larvae were microscopically identified to species level using existing identification keys [29]. Larval sampling was followed on weekly basis unless stated otherwise.

### Identification and characterization of plant cover types

This was a preliminary study done to determine whether plant cover type had any impact on the abundance of mosquito larvae in habitats within the Nyalenda study site. Plants growing within suitable mosquito breeding habitats were identified and categorized into two broad groups: those which grew along banks of water channels and those which grew inside the water channels. Plants which grew along the banks of the water channels were grouped as food and non- food crops. Plants which grew inside water channels with roots anchored in the soil were classified as rooted emergent plants while those suspended on the water surface were grouped as floating types.

### Identification and mapping of traditional water management practices

Four habitat types associated with different traditional water management practices were identified and incorporated in the design of this study. These were pools (on average 0.7 m deep × 2.1 m in diameter), small water canals (15 m × 1 m × 0.3 m deep), paddies (15 m ×15 m ×0.5 m deep) and swamps used for control (5 m × 15 m ×0.3 m deep). Except for the swamps (= control), all habitats were man-made. Sampling of mosquito larvae was done on Tuesday and Friday mornings for thirteen weeks (February to May 2008) based on the procedure used under plant cover habitats. The presence or absence of plant cover of each habitat was also recorded and mosquito larvae sampled according to the procedure above.

### Establishing the effect of plant cover type on mosquito breeding

Vegetation cover types inside or along the banks of man-made water canals in Nyalenda were identified. These consisted of arrow root (*Maranta arudinacea*) growing along banks of water canals and inside water canals, sweet potatoes (*Ipomea batatas*) growing along banks of water channels, African couch grass (*Cynodon dactylon*) growing inside water channels, water ferns *(Azolla filiculoides) *growing on the water surface and open sites with no plant cover (control). Each type of habitat (2 m × 0.75 m × 0.3 m) was replicated five times. All habitats were irrigated by running water from large canals (20 m away). Silt was removed, edges of the habitats were slashed while weeds growing between the plants were uprooted on weekly basis. Each habitat type was separated from the other by 10 cm thick wall made up of soil/mud with a narrow inlet on the upper part to allow flow of water. Mosquito larval sampling was followed as described above.

### Manipulation of mosquito breeding habitats through shading

This study was conducted for a period of 17 weeks from March to June 2007. Thirty-six mosquito breeding habitats (1 m × 1 m × 0.5 m) were created by building a shallow dyke (0.2 m) around each habitat. Each of the four locally grown plant species Napier grass (*Pennisetum purpureum*), arrow root (*Maranta arudinacea*), papyrus reeds (*Cyperus *spp.) and rice (*Oryza sativa*) were planted in each habitat and replicated six times. One additional habitat of rice was introduced and left intact to determine if weeding had any effect on mosquito breeding and larval survival. Another series of habitats was left unplanted (control). The habitats filled naturally with water by seepage from groundwater or from rainfall. Weeding was done once per month in all habitats, except in the unweeded rice habitats, to remove un-wanted plant species that would cause unforeseen effects on the experiment. Larval sampling was conducted using the standard dipping method as described above.

### Biological control of mosquito larvae

#### Investigations of *Bti *and *Gambusia affinis *for larval control

This study was done for a period of eight weeks from November to December 2007. The main goal was to estimate the optimum number of fish and the quantity of *Bti *required for effective control of mosquito larvae. Six different treatments were randomly administered. These included *Bti *1 day, *Bti *3 days and *Bti *5 days (*Bti *was put in water, left to stay for 1, 3 and 5 days before larvae were introduced), *Bti *and fish, *Bti *only and fish only while one series was left untreated to act as a control. Each treatment was replicated 25 times. Small plastic washbasins (27.5 cm × 17.3 cm × 10 cm) filled with two litres of water collected from the Nyalenda field site to a depth of three cm were used. Sixty larvae consisting of 30 early (L1 and L2) and 30 late (L3 and L4) instars were randomly dispensed into each basin using a rubber pipette. Each basin containing water and larvae, were then randomly assigned the six treatments as shown above. In total, 9000 laboratory-reared larvae of *An. gambiae s.s*. were used. The optimum *Bti *dosage and concentration of 5 mg/l of water was determined based on the existing literature [30]. Preliminary trials were done with different numbers of adult fish, which were offered 60 larvae (mixed larval stages of development) and we found that four adult fish were able to consume 60 larvae in 24 h. Different sizes of mosquito fish were used to cater for differences in predation resulting from effect of size. The number of live larvae present after introduction of the treatments in different wash basins was recorded after 24 and 48 h of exposure.

### Biological control of mosquito larvae under field conditions

This study was done for a period of 13 weeks from February to May 2008. Thirty man- made habitats (1 m × 1 m × 1 m) were created as mosquito larval habitats by building a 30 cm shallow dyke around each habitat. Six treatments were randomly administered as follows, *Bti, Bti *+ fish in full required amount, *Bti *+ fish at half the required amount of each, fish introduced once, and fish introduced fortnightly, while one habitat series was left untreated to act as a control. Each treatment was replicated five times. In the man-made habitats provided with fish, an extension hole measuring 30 cm × 30 cm × 30 cm was dug at the bottom to provide a hiding place for the fish whenever the water reduced to minimal levels, in order to avoid potential deaths resulting from dehydration. The average quantity of *Bti *applied was determined by calculating the volume of water present in the site before treatment was administered. Granule formulation of *Bti *was broadcasted into each sampling site at the rate of 5 mg/l of water. The total number of mixed sizes of mosquito fish (4 to 7 cm) used was based on the feeding rate of four mosquito fish per 60 mosquito larvae per day. This was also used as the minimum number of mosquito fish applied in the respective habitats. Treatments were repeated at 14-day intervals, each time on the same day of the week, except for habitats that had predatory fish introduced only-once.

A similar procedure was used for biological control of mosquito larvae within man-made water canals with different vegetation cover types. Six treatments were randomly administered in canals habitat with open water (control), *Azolla *growing on the water surface, sweet potatoes and arrow roots growing along the banks of water channels, African couch grass and arrow roots growing inside the water canals. Larval sampling of mosquitoes was done 24 h after treatment and thereafter regular sampling of mosquito larvae was conducted twice weekly using the standard dipping method as described above.

### Data analysis

Data analysis was done using SPSS 15.00 for windows (SPSS Inc, Chicago, IL, USA). The General Linear Model (GLM), multivariate analysis was used to calculate the estimated marginal means for larval densities. Generalized Linear Model (GLM), with probability for normal distribution and log linked function was used for calculation of Odds ratio and comparison of larval densities within different habitats with the control. Only anopheline larval data was included in the analysis.

## Results

### Water management practices and larval abundance

Anopheline larval abundance sampled from the pools, paddies and water canals was compared with the control (swamp). The abundance of early instars was significantly different in water canals (P < 0.05) and pools (P < 0.05). Early instars were twice more likely to be sampled in pools (OR 2.328, 95% CI 1.057-5.124) and water canals (OR 2.512, 95% CI 1.151 - 5.482) than in the swamps. Water management practices had no significant (P > 0.05) influence on the abundance of late instar anophelines. However, late instars were twice more likely to be found in pools (OR 2.519, 95% CI 0.281- 22.610) and four times in water canals (OR 4.240, 95% CI 0.521 - 34.478) when compared with the natural swamps.

### Vegetation cover and larval abundance

**Figure 1:**
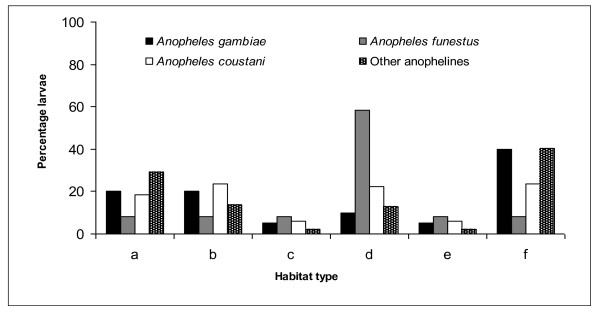
**Abundance of late instar larvae of anopheline species in habitats with: a) arrow roots growing in water, (b) arrow roots growing along water banks, (c) sweet potatoes along water banks, (d) couch grass in the water, (e) *Azolla *on water surface, and (f) control without plant cover, in Nyalenda**.

A total of 722 late instar larvae of anopheline mosquitoes were identified. Table 1 provides a list of plants common in the study area. A comparison of different habitats showed that open sites recorded the highest percentage of anopheline larvae (31.16%; n = 224) while those with arrow roots growing in water had 22.58% (n = 163), arrow roots growing along water banks 14.82% (n = 107), African couch grass growing inside water 14.54% (n = 105) and sweet potatoes growing along the water banks 13.85% (n = 100). The lowest percentage of anopheline larvae (3.05%, n = 23) was recorded where the water surface was covered by Azolla. The abundance of both early (OR 0.290, P = 0.001) and late instar larvae (OR 0.264, P = 0.025) of anophelines was reduced by 71 and 73%, respectively, in habitats covered with *Azolla *(Table 2). Although there were significant differences among habitats with other plant cover types (Figure 1), habitats with arrow roots recorded lower densities of both early and late instar larvae.

**Table 1:** Plant species grown in water and along water banks in Nyalenda

Category of plants	Plant species
**a) Plants grown along the water banks**	
(i) Food crops	*Zea mays, Phaseolus vulgaris, Phaseolus aureas, Elucine coracana, Sorghum sativum, Musa paradisiaca, Brassica *spp *(eg Kales), Colocasia esculenta, Manihot esculenta, Ipomea batatas, Lycopersicon *sp, *Saccharum officinarum, Cucurbita spp*.
(i) Non food crops	*Pennisetum purpureum, Digitaria scalarum, Cynodon nlemfuensis, Cyperus rotundus, Commelina *spp, *Ricinus communis*.

**b) Plants growing in water**	
(i) Emergent plants	*Colocasia esculenta, Digitaria scalarum*, *Cynodon nlemfuensis, Cyperus rotundus, Oryza sativa*,
(i) Floating plants	*Azolla filiculoides, Spirogyra *spp, *Rhodophyte *spp, *Phaeophyte *spp

**Table 2:** Abundance of early and late instar larvae of anopheline mosquitoes in man-made habitats covered with different plant covers

Early instars						Late instars				
**Parameter**	**EMM**	**95% CI of EMM**	**Odds ratio**	**95% CI for Exp (B)**	**P**	**EMM**	**95% CI of EMM**	**Odds ratio**	**95% CI for Exp (B)**	**P**

*Azolla*	0.450	0.064-0.964	0.290	0.144 - 0.584	0.001*	0.236	0.030-0.502	0.264	0.082 - 0.847	0.025*
Arrow roots inside water	1.343	0.829-1.857	0.866	0.491 - 1.526	0.620	0.757	0.491-1.023	0.848	0.535 - 1.343	0.482
Arrow roots on the water banks	1.557	1.043-2.071	1.005	0.619 - 1.631	0.985	0.657	0.391-0.923	0.736	0.446 - 1.216	0.231
Couch grass	1.486	0.972-2.000	0.959	0.595 - 1.545	0.862	0.829	0.563-1.095	0.928	0.599 - 1.437	0.738
Sweet potato outside	1.757	1.243-2.271	1.134	0.711 - 1.808	0.599	0.786	0.520-1.052	0.880	0.561 - 1.381	0.576
Control	1.550	1.036-2.064	1			0.893	0.627-1.159	1		

* shows larval densities that are significantly different from the control. EMM = estimated marginal mean CI = confidence interval; P = significance level at 95%.

*Anopheles gambiae *s.l. and *An. funestus *constituted 3.05% (n = 22) and 1.8% (n = 13), respectively, of all late instar larvae of anopheline mosquitoes identified from all habitats. *Anopheles gambaie *s.l. (36.36%, n = 8) were mostly sampled in the open habitats, while water surfaces covered with *Azolla *recorded the lowest number (4.55%, n = 1). Compared with other habitats, *An. funestus *were mainly recorded in habitats with African couch grass (53.85%, n = 7). *Anopheles coustani *Laveran, which has been reported recently as a possible vector species of malaria in East Africa [17] formed 28.81% (n = 208), while other anophelines that are non- vector species of malaria constituted 66.34% (n = 479).

### Manipulation of mosquito breeding habitats through shading

Young anophelines (L1 and L2) were abundant in all habitat types but the numbers of late stage larvae (L3 and L4) were fewer in most habitats except in the controls, weeded rice and habitats covered by Napier grass (Table 3). The densities of young anophelines were significantly reduced by 58% (OR = 0.414, P = 0.002), 51% (OR = 0.488, P = 0.038) and 42% (OR = 0.577, P = 0.051) in habitats with Napier grass, unweeded rice and arrow roots, respectively, when compared with control habitats. Late stage larvae were significantly reduced by 95% in habitats where arrow roots were grown (OR = 0.045, P = 0.004), and by 91% in habitats containing unweeded rice (OR = 0.091, P = 0.026), when compared with the control habitats (Table 3).

**Table 3:** Abundance of early and late instar larvae of anopheline mosquitoes in man-made habitats with different plant covers

Early instars						Late instars				
**Parameter**	**EMM**	**95% CI for EMM**	**Odds ratio**	**95% CI for Exp (B)**	**P**	**EMM**	**95% CI for EMM**	**Odds ratio**	**95% CI for Exp (B)**	**P**

Arrow roots	1.100	0.683-1.517	0.577	0.332-1.003	0.051*	0.006	0.036-0.048	0.045	0.005-0.380	0.004*
Unweeded rice	0.929	0.339-1.520	0.488	0.247-0.962	0.038*	0.012	0.047-0.071	0.091	0.011-0.755	0.026*
Weeded rice	1.353	0.763-1.943	0.710	0.387-1.303	0.269	0.094	0.035-0.153	0.727	0.227-2.334	0.593
Papyrus	1.976	1.386-2.567	1.037	0.621-1.731	0.889	0.035	0.024-0.094	0.273	0.052-1.442	0.126
Napier	0.788	0.371-1.206	0.414	0.238-0.718	0.002*	0.065	0.070-0.189	0.500	0.177-1.410	0.190
Control	1.906	1.316-2.496	1			0.129	0.023-0.107	1		

* shows larval densities that are significantly different from the control. EMM = estimated marginal mean CI = confidence interval; P = significance level at 95%.

**Figure 2:**
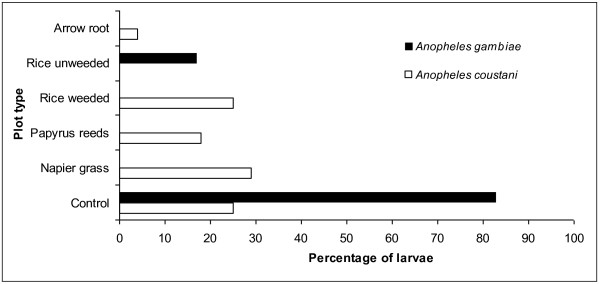
**Larval distribution of *Anopheles gambiae *and *An. coustani *in the control and habitats provided by different plant cover types, expressed as a percentage of the total number of larvae collected**.

Overall, anophelines comprised 29% of the total larval population sampled (N = 2445); while culicines (71%) were most abundant. Almost 85% of all *Anopheles gambiae *s.l. collected were from the control habitats while *An. coustani *was present in all habitats except the unweeded rice habitats (Figure 2).

### Efficacies of Bacillus thuringiensis var israelensis and Gambusia affinis for mosquito larval control

The percentage of larvae that was alive after 24 and 48 h of exposure to different treatments was quite low. Treatment with *Bti *recorded a few pupating larvae, but the resulting pupae were unable to develop into adults. Analysis of variance found significant differences among the treatments (F = 16.457; df = 4; P < 0.001). Pairwise comparison of different treatments showed that the number of larvae exposed to *Bti *and fish, *Bti *1 day, *Bti *3 days and *Bti *5 days were not statistically different (P = 1.0) from each other. However, apart from the control, treatment with fish recorded significantly more surviving larvae after 24 h when compared to those treated with *Bti *1 day, *Bti *3 days and *Bti *5 days old (P < 0.001).

### Efficacy of *Bti *and fish in man-made habitats

Anopheline larvae were sampled more from the control and in habitats with fish only (Table 4), whereas more culicine larvae (data not shown) were recorded in habitats treated with full amounts of *Bti *and fish. The effect of treatment type on young instars were significantly observed in habitats provided with *Bti *and fish, in half (OR = 0.650, P = 0.004) and full (OR = 0.325, P < 0.001) quantities of the required amount when compared to the control habitats. However, for the late instars, habitats with *Bti *and fish, half were marginally significant in comparison to the control whereas habitats provided with full quantities of *Bti *and fish were significantly different (OR = 0.344, P < 0.001) (Table 4).

**Table 4:** The distribution of early and late instars of Anopheline mosquitoes in man-made habitats (A = ponds, B = water canals) provided with different treatments

Early instars							Late instars				
**Variable**	**Parameter**	**EMM**	**95% CI for EMM**	**Odds Ratio**	**95% CI for Exp (B)**	**P**	**EMM**	**95% CI for EMM**	**Odds Ratio**	**95% CI for Exp (B)**	**P**

A) Ponds	*Bti *only	2.376	1.926-2.826	0.891	0.679 - 1.169	0.404	0.570	0.364-0.776	0.752	0.475-1.192	0.225
	Fish only	3.067	2.604 - 3.505	1.145	0.899 - 1.460	0.272	0.903	0.697-1.109	1.192	0.793-1.791	0.398
	*Bti *-Fish (half)	1.733	1.283-2.183	0.650	0.486 - 0.869	0.004*	0.485	0.279-0.691	0.640	0.405-1.012	0.056*
	Fish Once	2.667	2.217-3.117	1.000	0.786 - 1.272	1.000	0.964	0.757-1.170	1.272	0.844 - 1.916	0.250
	*Bti *-Fish (full)	0.867	0.417-1.317	0.325	0.211 - 0.499	0.000*	0.261	0.054-0.467	0.344	0.190 - 0.623	0.000*
	Control	2.667	2.217-3.117	1			0.758	0.551-0.964	1		

B) Water canals	*Bti *only	0.635	0.115-1.156	0.186	0.120-0.289	0.000*	0.161	0.042-0.365	0.137	0.068-0.278	0.000*
	Fish only	1.906	1.386-2.427	0.558	0.374-0.832	0.004*	0.547	0.344-0.750	0.465	0.288-0.750	0.002*
	*Bti*-Fish	0.740	0.219-1.260	0.216	0.143-0.327	0.000*	0.125	0.078-0.328	0.106	0.056-0.200	0.000*
	Control	3.417	2.896-3.937	1			1.177	0.974-1.380	1		

* shows habitats that are significantly different from the control. EMM = estimated marginal mean; CI = confidence interval; N = number of times sampled; P = significance level at 95%

In man-made canals, all treatment types were significantly different from the control (all P < 0.05). There was an overall reduction of 73.03% in the population of all larval stages of anopheline mosquitoes. Late instar larvae of anopheline mosquitoes were reduced by 87% (n = 173), 59% (n = 117) and 92% (n = 183) due to application of *Bti *only, fish only and *Bti *and fish, respectively. When compared with the control, late instars were reduced by 89% (OR 0.106, P < 0.001) and 86% (OR 0.137, P < 0.001) in habitats with *Bti *and fish, and those with *Bti *only, respectively. Generally, more larvae were recorded in both ponds and canals provided with fish as the only control option (Table 4).

**Figure 3:**
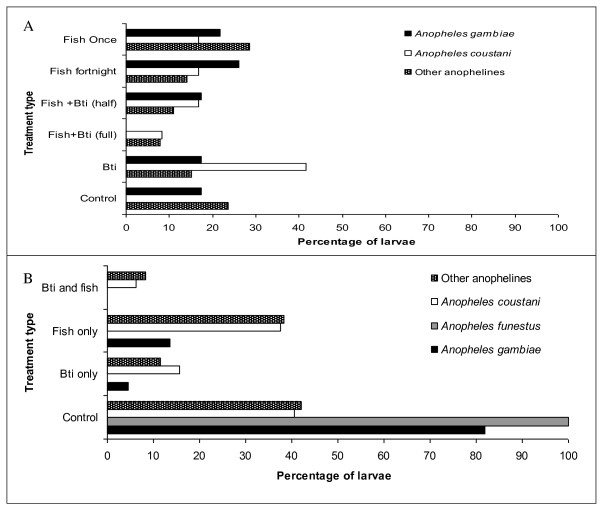
***Anopheles gambiae, An. funestus *and *An. coustani *larval distribution in the (A) ponds and (B) canal man-made habitats under different treatments expressed as a percentage of the total number of larvae recorded**.

*Anopheles gambiae *s.l. was recorded in all habitats except those provided with *Bti *and fish in full quantities, whereas more *An. coustani *were recorded from habitats containing *Bti *alone (Figure 3A). The population of *An. gambiae *reduced by 83.33% due to *Bti *only, 50% by mosquito fish, while both mosquito fish and *Bti *caused a reduction of 100%. *Anopheles funestus *was only recorded in control habitats (Figure 3B).

## Discussion

Simple strategies such as locally cultivated cover crops and plants to provide shade over mosquito breeding habitats as well as the use of predatory fish in combination with *Bti *are feasible options for the control of immature mosquitoes, including malaria vector species. All habitats provided with shade from *Azolla*, sweet potatoes, arrow root, Napier grass, rice and papyrus reeds supported significantly fewer anopheline larvae than the controls.

Habitats provided with shade did not have *An. gambiae *s.l. except for the unweeded rice, while *An. coustani*, a less important malaria vector, was recorded in all the habitats except in unweeded rice habitats. These effects exceeded our expectations, because *An. gambiae *s.l. was never recorded from treated sites except from unweeded rice, where it was found once. By contrast, *An. gambiae *was frequently present in unshaded control sites. *Anopheles funestus*, a malaria vector species of secondary local importance, was more sampled in canal habitats covered with couch grass. Longitudinal studies in Nyalenda established that *An. arabiensis *was the main species of the *An. gambiae *complex found in this area [5]. *Anopheles arabiensis *thrives best in open, sunlit transient habitats [29,31] hence shading potential breeding habitats might have affected its abundance.

There was an overall reduction in larval populations within habitats provided with cover crops or plants. As the crops grew taller, increase in height was directly proportional to shade over the mosquito habitat before the crop reached maturity and started to senesce. The findings are in agreement with other studies that found heavy shade to be negatively correlated with larval abundance of anophelines in breeding sites [2,32-35]. Previous studies carried out in Uganda showed that *An. gambiae *s.l. did not breed in the interior of papyrus swamps in their natural, undisturbed state [36]. In our study papyrus reed seedlings were transplanted into man-made habitats, hence they were not in their natural state and a reasonable abundance of both anopheline and culicine larvae were recorded within the habitats. The main malaria vector in western Kenya, prefers open, sunlit pools of water, however such habitats become unsuitable for ovipositing females when shade increases [6]. This is probably caused by the action of shade, which lowers the water temperature and reduces algal growth. Gravid female mosquitoes select to oviposit in sun-exposed sites [37].

The numbers of *An. gambiae *s.l. mosquito larvae recorded 24 h and 48 h after exposure to treatment was significantly influenced by treatment type. However, man-made habitats provided with both *Bti *and fish resulted in greater reductions of anopheline larval population densities when compared to habitats where only *G. affinis *was introduced. These results are comparable to the outcome of experiments conducted by Blaustein [38] where *G. affinis *alone failed to control mosquitoes in experimental rice habitats. These results indicate that the predatory effectiveness of mosquito fish on anopheline mosquito larvae diminished when introduced into the man-made larval habitats. The contrast in the findings could be attributed to other factors that we did not investigate/foresee, such as fish preying on other aquatic organisms, external food or invertebrate sources and physical factors such as turbidity. Homski et al. [39] found that higher turbidity in man-made habitats may have favored a higher abundance of invertebrates and reduced visibility of anopheline larvae for mosquito fish than in sites covered with emergent vegetation. In addition, under natural circumstances other fish species may be better predators on anopheline larvae [40]. The findings on larval control options suggest that *G*. *affinis *and *Bti*, when used together in the right quantities complement each other and are more effective in reducing mosquitoes in man-made habitats.

In this study, *Bti *was applied in habitats once in a fortnight, which matched with larviciding studies, carried out in Eritrea [19]. With a two weeks interval, our results show a low impact of *Bti *only on larval abundance. However studies by Fillinger and Lindsay [16] and Majambere *et al*. [18], report microbial larvicides such as *Bti *to have greater efficacy (95%) when applied to anopheline larval habitats in optimum quantities on a weekly basis. If weekly application of *Bti *would have been followed, then habitats provided with *Bti *only may have been as effective as those provided with *Bti *and fish on larval abundance. In addition, the persistence of *Bti *endotoxins in our study may have reduced rapidly under field conditions, hence showing no apparent effect on anopheline larval abundance. As previous studies clearly showed that *Bti *is non toxic to non-target organisms [16,30], we used this property of *Bti *to serve as a basis for integrating this product with *G. affinis *for increased efficacy of larval control.

The trials in this study were done under field conditions in man-made habitats that were naturally colonized by mosquito larvae. Under these conditions, external factors were not controlled and could have played an important role in the colonization and growth of mosquito larvae in the respective habitats. The variations in water level and occasional flooding of habitats could not be avoided, as the sites were exposed to ambient conditions. Factors such as water turbidity, nutrient content in water, cannibalism, predation of immature stages, parasitism, pathogens, competition, water temperature and plant odours that could have either repelled or attracted female mosquitoes during oviposition [41-47] were not controlled and hence could have played a role in the results obtained. All larval stages of culicine mosquitoes increased as vegetation cover increased progressively from man-made ponds, small water canals, rice paddies to swamps. Habitats with few anopheline larvae recorded more culicine larvae, while those that recorded more anophelines had fewer culicine larvae. This suggests selective oviposition behaviour among these mosquito families [37,48,49]. In Nyalenda, the water present in breeding habitats was often polluted with debris and human waste, which might have favored proliferation of culicine mosquitoes (data not shown) and at the same time water quality may have had a negative impact on the efficacy of the treatments provided. Competition and differences in the physical-chemical characteristics of the water may have played a role in structuring larval populations, although these factors were not investigated in this study. The standard dipping method was used to estimate mosquito larval densities, which may have underestimated larval abundance [28,50,51] and consequently, may have influenced the amounts of *Bti *and numbers of *G. affinis *used, leading to contrasting results.

In western Kenya, areas that were previously natural swamps and forests have been transformed into agricultural fields mainly due to human population pressure [2]. These agricultural developments have an impact on the ecological characteristics of the local mosquito vector in terms of density, local microclimate and malaria incidence [2,33,52,53]. Communities in western Kenya are willing to take part in malaria control [27] and to effect this, simple control strategies suitable for the local of mosquito vectors need to be available as a way of adapting to the changes in land use. Results from this study indicate that locally available leafy plants could be used for mosquito control especially in areas under traditional agriculture. Use of edible fish [40] and mosquito fish are other options that can easily be put into practice, especially in areas where water is always present.

The effectiveness of biological larvicides for the control of African anophelines has already been demonstrated by several studies [16,17,54] and in areas where locally available solutions are not feasible and where water cannot be drained, then application of microbial larvicides could be the best option. Although our results are spatially and temporally limited, the option of using shade from locally available crops and predatory fish seems an easily applicable alternative for the control of mosquito larvae. More importantly, as the level of morbidity resulting from the specific problem of malaria is a net result of a balance between livelihood and ecosystem factors [55] an ecohealth approach to malaria control is bound to produce discernable and long lasting effects.

This study was part of an ongoing project in different agro-ecological settings in two highland villages (Lunyerere and Fort Ternan) and one peri-urban area (Nyalenda), where most larval habitats were man-made [5]. The field studies reported in this paper were done in the peri-urban area of Kisumu town to assess the best options of controlling immature mosquitoes. For Nyalenda, larviciding and use of predatory fish seem promising and can be supplemented with the existing adulticiding options.

## Conflict of interest statement

We declare that we have no conflict of interest. This work was part of the project funded by the Dioraphte Foundation, The Netherlands. The funding organization had no role in the analysis or interpretation of the results or in the drafting of the manuscript.

## Authors' contributions

SSI and CKM designed study, carried out data collection and writing of the manuscript. SSI performed the statistical analysis. WT and RW assisted with study design and in editing of the manuscript. All authors read and approved the final version of manuscript.
